# Fabric-Based Electrochemical Glucose Sensor with Integrated Millifluidic Path from a Hydrophobic Batik Wax

**DOI:** 10.3390/s23135833

**Published:** 2023-06-22

**Authors:** Isa Anshori, Elfrida Vanesa Heriawan, Putri Yulianti Suhayat, Dedy H. B. Wicaksono, Samuel Priyantoro Kusumocahyo, Ardianto Satriawan, Wervyan Shalannanda, Latifa Dwiyanti, Casi Setianingsih, Murni Handayani

**Affiliations:** 1 School of Electrical Engineering and Informatics, Bandung Institute of Technology, Bandung 40132, Indonesiawervyan@telecom.stei.itb.ac.id (W.S.);; 2 Research Center for Nanosciences and Nanotechnology (RCNN), Bandung Institute of Technology, Bandung 40132, Indonesia; 3 Department of Biomedical Engineering, Faculty of Life Sciences and Technology, Swiss German University, Tangerang 15143, Indonesia; 4 Department of Chemical Engineering, Faculty of Life Sciences and Technology, Swiss German University, Tangerang 15143, Indonesia; 5 Department of Computer Engineering, School of Electrical Engineering, Telkom University, Bandung 40257, Indonesia; 6 Research Center for Advanced Materials—National Research and Innovation Agency (BRIN), Tangerang Selatan 15314, Indonesia

**Keywords:** millifluidic devices and lab-on-chip devices, chemical and biological sensor, fabric-based, continuous glucose monitoring, sensor testing and evaluation, stencil printing

## Abstract

In recent years, measuring and monitoring analyte concentrations continuously, frequently, and periodically has been a vital necessity for certain individuals. We developed a cotton-based millifluidic fabric-based electrochemical device (mFED) to monitor glucose continuously and evaluate the effects of mechanical deformation on the device’s electrochemical performance. The mFED was fabricated using stencil printing (thick film method) for patterning the electrodes and wax-patterning to make the reaction zone. The analytical performance of the device was carried out using the chronoamperometry method at a detection potential of −0.2 V. The mFED has a linear working range of 0–20 mM of glucose, with LOD and LOQ of 0.98 mM and 3.26 mM. The 3D mFED shows the potential to be integrated as a wearable sensor that can continuously measure glucose under mechanical deformation.

## 1. Introduction

The detection of the level of analytes, for example, glucose, lactate, and urea, in people with related diseases are essential for their health. For instance, diabetic patients require constant monitoring of glucose levels. Diabetic patients need to monitor their blood sugar levels to manage their treatment plan and to prevent long-term complications of diabetes. Blood sugar monitoring determines when insulin is needed to reduce blood glucose levels in the body or vice versa when additional glucose is needed to raise blood glucose levels [1]. A conventional technique for determining analyte concentration, in this case, blood glucose level, includes an intermittent drawing of blood, placement to the test strip, and lastly, determination of the blood glucose level itself, which uses various methods: colorimetric, electrochemical, or photometric. Many patients find periodic testing inconvenient, and sometimes they can forget to do the monitoring test, so the demand for longer real-time measurements is high. Although non-enzymatic approaches via noble metals have been widely tested, conventional enzymatic-based sensors remain the commonly available products in the market as the preference on its selectivity [2].

Trend in developing continuous biosensors has been rising in the recent decade in the form of wearable or flexible sensors. This trend follows the success of wearable devices in the form of smartwatches, capable only of measuring biosignals. Wearable biosensors have been successfully realized using the techniques of colorimetry and electrochemistry. Colorimetric devices were developed based on enzymatic and non-enzymatic based. The analyte-induced colorimetric change had been developed in some studies using enzymes, such as glucose oxidase and lactate oxidase, with inspection by simple visual observation or using a smartphone for concentration measurement with some enhancement routes obtained through integrating catalytic nanomaterials [3,4,5,6,7,8,9]. Non-enzymatic-based colorimetric sensors were commonly developed by utilizing nanomaterials [10,11]. However, obtaining accurate measurements based on the colorimetric technique is still less sensitive than electrochemical-based flexible/wearable sensors.

The development of a fabric-based electrochemical device (FED) is a new cheaper alternative to electrochemical flexible/wearable sensors that can be used for continuous monitoring of analytes. FEDs were developed based on enzyme-used [12,13,14,15,16,17] or enzyme-free [12,18] techniques. Performance enhancement is also typically obtained by incorporating nanomaterials. Cotton fabric can be used as a substrate material for this electrochemical device due to the favorable properties of the textile. FED is lightweight, flexible, durable, widely available in the market at a considerably low price, and has a wicking property, facilitating the passive attraction of analytes solution to the sensors. Moreover, further development of this proposed design can easily be integrated into clothes as a wearable sensor. It is also envisioned for real-time analysis of physiological conditions to monitor patients who need constant monitoring. One of the widely used methods for measuring and monitoring analyte concentration is through a screen-printed-based biosensor.

Screen-printed electrodes for biosensing have been one of the most promising approaches towards a simple and rapid production of biosensors [19]. Producing reproducible, disposable, and stable screen-printed electrodes had a crucial impact on electrochemical biosensor development. It is one of the most popular technologies used in point-of-care applications mainly because of its innate design in which its ink can be easily modifiable. The reference, working, and counter electrodes in screen-printed technologies made this technology adaptive and accurate [20]. Electrochemical sensors fabric-based electrochemical device (FED) developments by combining stencil printing and wax transferred have been a promising approach for mass-scale and lower the cost of production [21]. Another technique of coating the yarn with conducting ink before being handloom-woven as electrodes has also been tested, although the need for ink volume should be higher than the stencil approach [22,23].

In this study, we evaluated the sensor design from Malon et al.’s previous work and modified the glucose detection system [24]. The stencil printing technique was selected due to the easiness of the fabrication method and can be directly used with commercial conductive ink products. Using this strategy, a hydrophobic batik wax structure was defined for creating the sensing chamber, and we tested its possibility as a fluidic channel. We also evaluated its sensing capabilities under several mechanical and wicking tests to understand its effect when the system is integrated into clothing.

## 2. Experimental

### 2.1. Equipment and Materials

The equipment used in this research includes a Silhouette Cameo 3 digital cutter interfaced with Silhouette Studio software (Silhouette America, Lindon, UT, USA), Dynamic 330 laminating machine, oven, and two-axis linear stage. All electrochemical measurements were performed using Emstat2 potentiostat interfaced with PSTrace software (Palmsens BV, Houten, The Netherlands).

The main fabric material used for this mFED was white mori cotton fabric (fabricated using Ne1 40s yarn count). D-(+) Glucose ($C_{6}H12O_{6}$), sodium carbonate ($Na_{2}CO_{3}$), disodium hydrogen phosphate ($Na_{2}HPO_{4}$ anhydrous), dihydrogen phosphate, ($KH_{2}PO_{4}$ anhydrous), sodium chloride (NaCl), potassium chloride (KCl), and sodium hydroxide (NaOH) were purchased from Merck. Glucose oxidase/peroxidase reagent (G3660-1CAP) was purchased from Sigma Aldrich. Electrode paste carbon modified with Prussian Blue (C-PB) (C2070424P2) and silver/silver chloride (Ag/AgCl) (C2130823D1) paste were purchased from The Gwent Group. Chemistry control level I (lot number: 717401) and level II (lot number: 609902 were purchased from Pointe Scientific, Inc., Canton, MI, USA. Solutions were prepared using deionized (demineralized) water (27256) purchased from HACH Company, Loveland, CO, USA.

The electrolyte used in this experiment was PBS 0.1 M, prepared using an appropriate dilution of PBS 1.0 M (pH 7.4). A stock solution of 50 mM glucose standard solution was made in PBS 0.1 M, and more dilute glucose standards (2–20 mM) were prepared by appropriate dilution in PBS 0.1 M. Glucose oxidase (500 U)/peroxidase reagent was diluted in 39.2 mL deionized water.

### 2.2. Fabrication of mFED

#### 2.2.1. Design of mFED with Hydrophobic Batik Wax Fluidic Pattern

**Figure 1:**
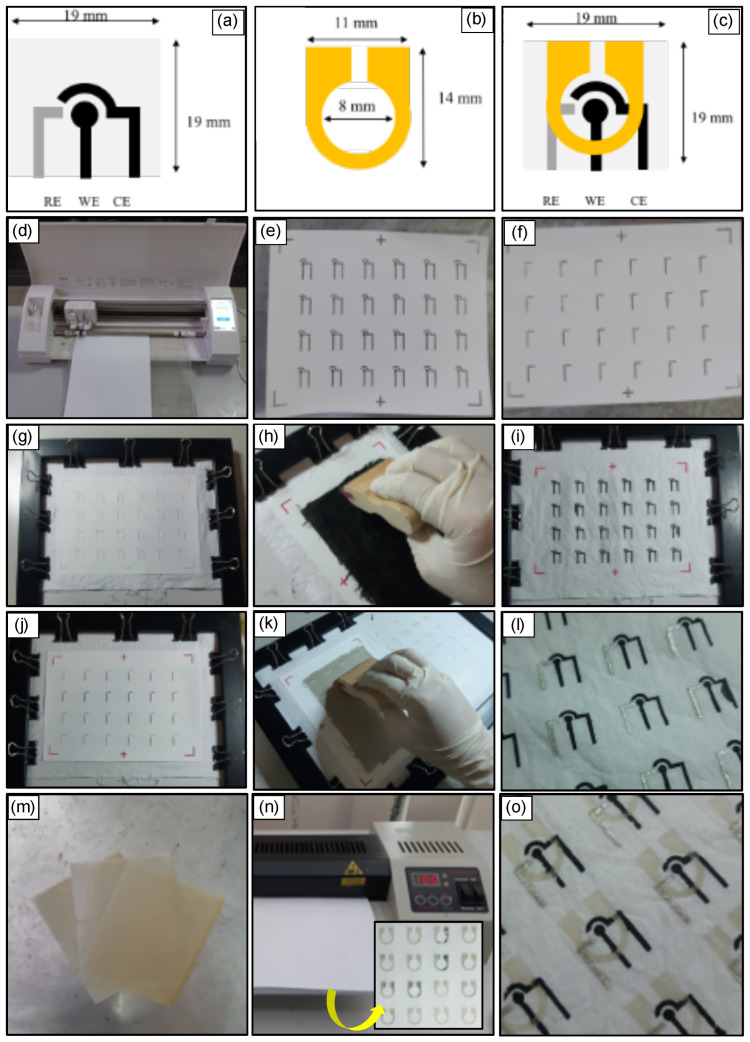
Schematic illustration of the mFED fabrication: (**a**) Cotton fabrics electrode consists of reference electrode (RE), working electrode (WE), and counter electrode (CE). (**b**) Wax pattern to create the fluidic chamber, covering the area of RE, WE, and CE. (**c**) Full mFED design. (**d**) Vinyl sticker attached to a mat of a digital cutter. (**e**) Vinyl sticker cut for C-PB stencil. (**f**) Vinyl sticker cut for Ag/AgCl stencil. (**g**) C-PB stencil was placed on top of scoured cotton fabric. (**h**) Applying C-PB ink. (**i**) Cotton fabric with C-PB ink pattern. (**j**) Ag/AgCl stencil was placed on top of scoured cotton fabric. (**k**) Applying Ag/AgCl ink. (**l**) Cotton fabric with Ag/AgCl and C-PB ink patterns. (**m**) Paper coated with wax. (**n**) The fluidic chamber stencil was sandwiched between cotton fabric (with Ag/AgCl and C-PB patterns) and wax-impregnated paper. The wax pattern was transferred to the cotton fabric by using a hot laminator. (**o**) The finished batch of mFED.

The template for patterning electrodes consisting of the working electrode (WE), reference electrode (RE), and counter electrode (CE) was designed using Silhouette Studio software (Figure 1a). First, the template for patterning the reaction zone was printed using a digital craft cutter on batik wax-impregnated paper (Figure 1b). Next, the wax-impregnated paper was placed on the mFED, and the laminating machine was used to transfer the wax onto the cotton fabric. The final setup is shown in Figure 1c.

#### 2.2.2. Stencil Printing Patterning

Fabrication of electrodes was conducted by using the stencil as the main material. The cotton fabrics were first scoured using anhydrous sodium carbonate ($Na_{2}CO_{3}$) to produce hydrophilic cotton fabric. Then, 1 L of deionized water was boiled until it reached 100 °C. Then, 20 g of $Na_{2}CO_{3}$ was dissolved into the solution. Cotton fabrics were soaked in the boiling solution for 10 min. After the treatment, the cotton fabric was rinsed with deionized water until the pH reached a neutral range (pH 6–7). The scouring process was done to remove the natural wax in the cotton fabrics, increasing hydrophilicity [25].

Next, Ag/AgCl stencil and C-PB stencil were made by cutting the self-adhesive vinyl paper using the digital cutter (Figure 1d). Both stencils have alignment marks cut on them for the precise result of electrode fabrication. There were two stencils for electrode patterns: C-PB (Figure 1e) and Ag/AgCl (Figure 1f). The scoured cotton fabric was then clamped between a photo frame and clipped using binder clips to keep it in place during ink transfer (Figure 1g,j), followed by pasting the respective inks using a squeegee (Figure 1h,k) to obtain the electrode pattern of C-PB (Figure 1i) and Ag/AgCl (Figure 1l), respectively. We created simple alignment patterns on the edge side, surrounding the stencils. After the electrode paste transfer was completed, the mFED was cured at 60 °C for 30 min in the oven.

We prepared an A4 paper (80 grams) separately and dipped it into the melted wax. After the paper was fully covered with the wax, it was picked up using a tweezer and let dry at room temperature before use (Figure 1m). Next, a wax pattern stencil was prepared using the digital cutter. The wax patterning technique was performed by stencil assistance sticking onto cotton fabric (with electrode patterns) and using a hot laminator.

Laminating machine with a temperature of 100 °C was used to transfer the wax onto the cotton fabric electrode. The stencil was used for the wax-impregnated paper to prevent the wax from seeping into the fabric during heat treatment. The steps were repeated for the backside of the electrode to ensure the wax penetrated to the other side of the cotton fabrics, making a leak-proof reaction zone and perfect hydrophobic fluidic path. Finally, the stencil and wax-impregnated paper was removed, allowing it to cool at room temperature. The device was ready to use after cutting the mFED into 19 × 19 mm (Figure 1c,o).

### 2.3. Electrochemical Measurement of the Device

#### 2.3.1. Characterization of Electrochemical Detection

**Figure 2:**
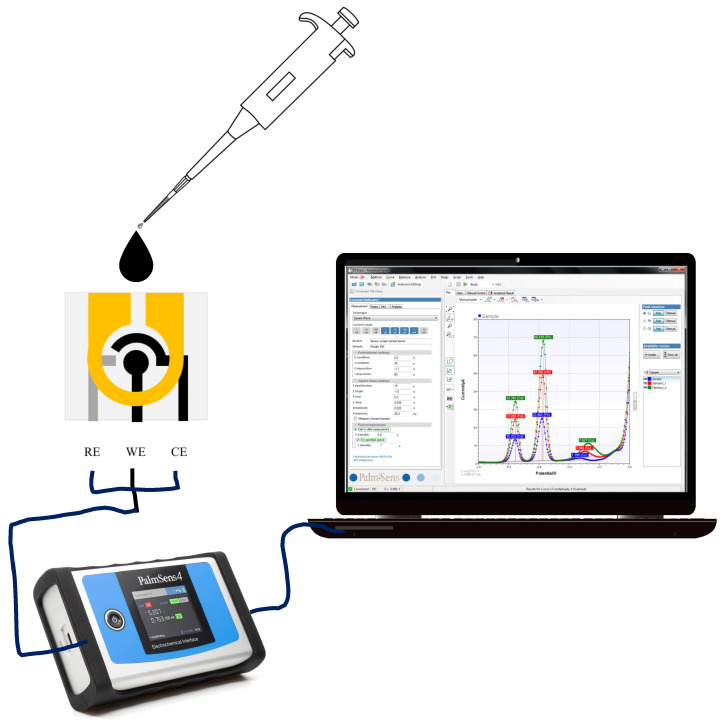
Illustration of the electrochemical measurement setup.

Electrochemical characterization of the C-PB electrode was done using the cyclic voltammetry method to study the electron transfer kinetics and redox process. The mFED was connected to a potentiostat using the provided crocodile clips (Figure 2). The cyclic voltammetry was performed by sweeping the potential range from −0.5 to 0.5 V, in which the redox reaction occurs. A total of 6 μL of 0.1 M PBS was deposited on top of the sample placement zone, and cyclic voltammetry was performed at potential scan rates ranging from 0.1 to 0.0025 V/s. The relationship between anodic–cathodic current peaks and the square roots of the scan rates was evaluated.

#### 2.3.2. Optimum Detection Potential for Glucose Sensing Application

This enzymatic electrochemical biosensor relies on detecting the hydrogen peroxide ($H_{2}O_{2}$) to quantify the level of analytes concentration. The method used for detecting $H_{2}O_{2}$ in this device is the cathodic reaction of $H_{2}O_{2}$ with the help of the carbon-Prussian blue electrode as the catalytic redox mediator. C-PB was chosen because it can be easily incorporated into the fabric-based electrochemical device, and the price is categorized as inexpensive compared to the most commonly used mediator, which is using platinum.

For the glucose measurement, 30 μL of the glucose oxidase enzyme solution (0.38 unit) was preloaded at the reaction chamber of the device and allowed to dry at room temperature for 30 min before measurement. The characterization for choosing the optimum potential was conducted using the cyclic voltammetry method. The C-PB electrode was first characterized in the absence of $H_{2}O_{2}$ (using 0.1 M PBS) and in the presence of $H_{2}O_{2}$ (using 50 mM glucose) at potential scan rates of 0.005 V/s. The current value in the absence of $H_{2}O_{2}$ and the presence of $H_{2}O_{2}S$ were plotted as a function of potential. Subsequently, the signal-to-background ratio (S/B ratio) was calculated to study the effect of the applied potential on the C-PB electrode. The highest signal-to-background ratio was chosen for optimum detection potential for further studies.

### 2.4. Analytical Performance

Chronoamperometry measurement was performed in different glucose concentrations ranging from 0 to 20 mM to measure the sensitivity of the mFED in detecting glucose. The chronoamperometry method was selected for this enzyme-based sensing application because it offers accuracy, better sensitivity, and a lower detection limit than the cyclic voltammetry method. The anodic currents produced in response to the changes in concentration were evaluated by plotting the linear calibration.

#### 2.4.1. Analytical Validation

The device’s accuracy was evaluated by demonstrating chronoamperometric measurements using a real sample from standard human serum. The chronoamperometric measurements were conducted using human serum levels I and II as the sample. The chemistry control serum was prepared by dissolving it in 5.0 mL of deionized water. The chemistry control serum level II was diluted with deionized water with a ratio of 1:1. A total of 6 μL of the sample was pipetted onto the glucose mFED before measurements. The results obtained from the mFED were compared with measurements using a glucometer.

#### 2.4.2. Continuous Measurement Using 3D mFED

**Figure 3:**
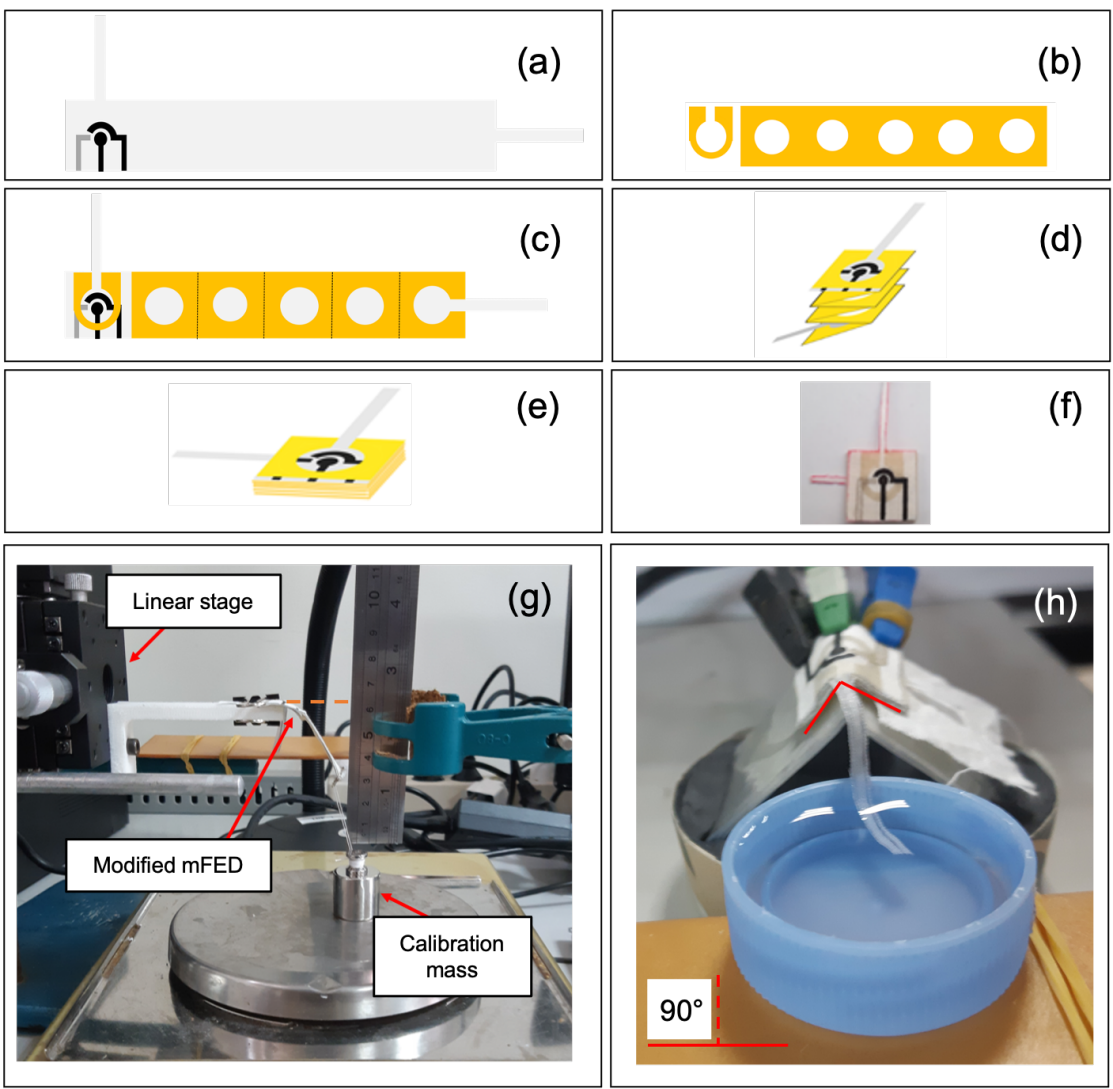
Fabrication of the continuous mFED platform and its evaluations. (**a**) Electrode patterns on fabric. (**b**) Wax pattern to be transferred to fabric with electrode patterns. (**c**) Final design. (**d**,**e**) Folding step in an accordion fashion. (**f**) Fabricated mFED for continuous measurement. (**g**) Mechanical strain test setup for bending movement. (**h**) For continuous measurement under folding strain.

**Figure A1:**
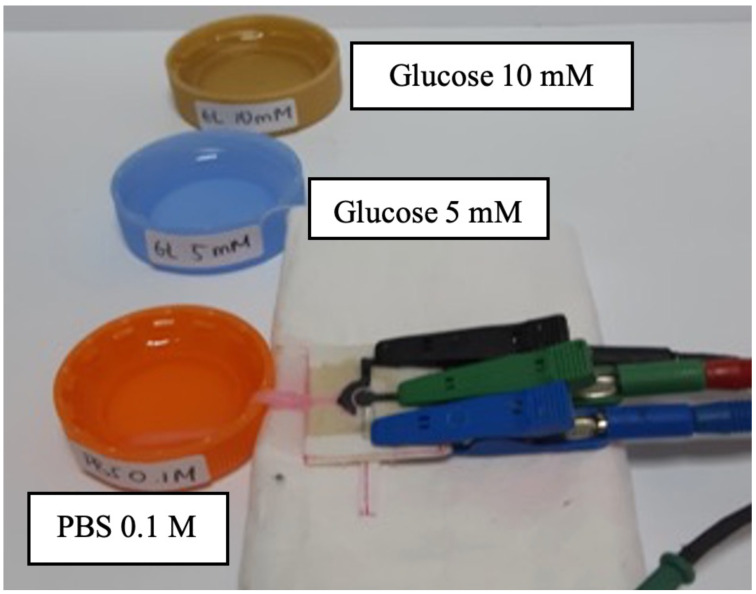
Measurement setup for chronoamperometry analysis.

Our goal is to develop a continuous mFED platform for real-time sample delivery and continuous analyte assessment using electrochemical measurement. The fabrication process used a similar stencil printing method as the non-continuous one (Figure 1). The only difference was the size of the cotton fabric (Figure 3). The size of the electrode fabric before it was folded was 19 × 114 cm (Figure 3a). This continuous platform will have a sample inlet extending away from the reaction chamber (2 × 30 mm) and a sample outlet (2 × 10 mm) for sample delivery. After transferring wax on the fabric (Figure 3b,c), the device was then folded into multiple layers in an accordion fashion (Figure 3d–f). For continuous dynamic measurement, chronoamperometry measurements were conducted using 3D mFED for 170 min. Figure A1 in the Appendix C section shows the measurement setup. There are three container solutions used in this evaluation: 0.1 M PBS, 5 mM glucose, and 10 mM glucose.

For the first 20 min, the mFED inlet was dipped into a reservoir containing PBS 0.1 M. The inlet was introduced to a new glucose solution with a concentration of 5 or 10 mM alternately every 30 min. The outlet of the continuous platform was placed on top of the cotton fabric to speed up the sample delivery. This experiment was conducted to evaluate the efficiency of sample delivery of the 3D mFED and to demonstrate the capability of the mFED to provide real-time monitoring of blood glucose for a certain period. The experiment was repeated three times using different mFEDs. Continuous chronoamperometry measurement was conducted for 3 h using chemistry control level 1 as the sample.

### 2.5. Mechanical Deformation Test

Potential suitability of this mFED to be integrated as a wearable sensor was tested by applying mechanical deformation to the mFED. The effect of mechanical strain on the mFED electrochemical performance was examined through amperometric detection of glucose under bending and folding movement. The first experiment is illustrated in Figure 3g, which shows the mechanical strain test setup for bending movement. The experiment was conducted by attaching an L-shaped bracket to a linear stage. One end of the mFED was clamped to the L-shaped bracket’s edge, and another mFED’s end was attached to a calibration mass and placed on top of an analytical balance. As the linear stage moves upward, it causes the mFED to bend and create a deflection that can be measured. The force applied to the mFED can be calculated from the mass measured on the analytical balance [26].

The second experiment was conducted by folding the mFED into 45° and 90° angles (Figure 3h). The mFED was attached to a mica plastic sheet fixed and shaped into 45° and 90° angles. The chronoamperometry was conducted during the deformation of the mFED. The potential deformation that a wearable sensor can undergo can be mimicked by performing these experiments. Chronoamperometric measurement was conducted for six hours (360 min) on flowing glucose solution at 5 mM concentration for each deflection point. Equations to calculate the strain were included in Appendix A.

In the current study, we focused on the preliminary development of the fabric sensor platform with a fluidic and sensing area created from a hydrophobic wax pattern. The sensor’s selectivity is supported by the usage of glucose oxidase enzyme that can specifically convert glucose to hydrogen peroxide and D-glucono-δ-lactone [27]. The interference studies were not conducted in the current work.

## 3. Results and Discussion

### 3.1. Fabrication of mFED

Two electrode paste transfer methods were performed in this thesis work. The first electrode transfer method used the paintbrush method [24]. As shown in Table 1, electrode fabrication using the paintbrush method has a high standard deviation; each device differs. Such results can happen because the paintbrush method is a low automation method; the result of each device depends on the hand of the one who fabricates the device and the pressure given while brushing the electrode paste onto the cotton fabric. As a result, the amount of electrode paste brushed onto each device is different and cannot be controlled. On the other hand, the amount of electrode paste used in this method is low because there was no excess electrode paste wasted in the process.

The brushing process of the electrode paste was not easy since the penetration of the electrode paste to the backside of the fabric was difficult using the paintbrush method. When the electrode paste does not penetrate to the other side, only on the surface of the cotton fabric, it can produce a high resistance value on the electrode itself. Our new electrode paste transfer method was developed using stencil and squeegee as tools for electrode paste transfer. A 5 cm squeegee forces the electrode paste to fill the stencil openings between the cut sticker paper and the cotton fabric substrate. The amount of electrode paste throughout the electrodes in the same batch is more evenly distributed using the stencil printing method. The rubber blade of the squeegee can also scrape the excess ink on top of the sticker paper to be dragged across the opening and force the ink to penetrate the cotton fabric substrate. For these reasons, a lower resistance value and lower standard deviation can be achieved (Table 1). Batch production also can be produced in an efficient amount of time. Table 2 summarizes results from the two methods of electrode transfer.

### 3.2. Electrochemical Measurement of the Device

#### 3.2.1. Evaluation for Finding Optimum Detection Potential

**Figure 4:**
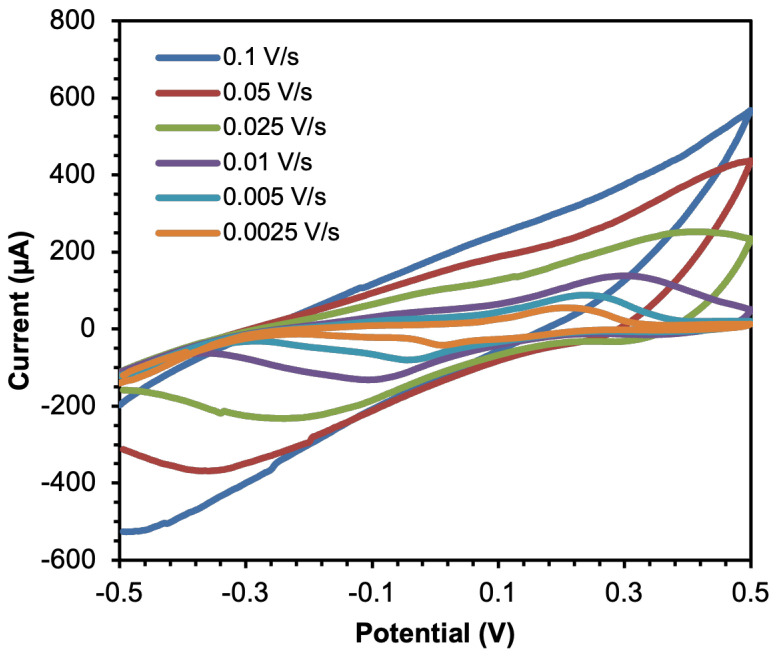
Cyclic voltammograms of C-PB electrodes on various scan rates in 6 μL 0.1 M PBS.

The cyclic voltammetry measurements were performed on the mFED within the potential limits of −0.5 to 0.5 V on various scan rates (0.1 to 0.025 V/s) in 6 μL 0.1 M PBS. Figure 4 shows the CV of C-PB responding to PBS. The result reveals that the reduction and oxidation (redox) reactions do not occur properly, and tend to present inert response reactions, although we increase the scan rate. Peak currents disappear and only expand the integrated area.

Next, we evaluate the performance of the device toward glucose determination. The basic principle of this biosensor is based on the enzymatic reaction of the glucose oxidase enzyme and β-D-glucose. Glucose oxidase (GOx) is used in most glucose biosensors in the market because of its high selectivity for glucose. β-D-glucose, in the presence of oxygen ($O_{2}$) and water ($H_{2}O$), will be oxidized into gluconic acid and hydrogen peroxide by the immobilized GOx on the mFED (Equation (1)). Glucose oxidase needs flavin adenine dinucleotide (FAD) as a redox cofactor, then FAD is reduced to ${\mathrm{FADH}}_{2}$ [28] (Equation (2)). Prussian blue is excellent as an $H_{2}O_{2}$ catalyst. Prussian blue (PB), $Fe^{\mathrm{III}}[Fe^{\mathrm{II}}{\left(\mathrm{CN}\right)}_{6}{]}^{1-}$, is reduced to Prussian white (PW), ${Fe^{\mathrm{II}}\left[Fe^{\mathrm{II}}{\left(\mathrm{CN}\right)}_{6}\right]}^{2-}$, in the presence of $H_{2}O_{2}$, and this produced hydrogen peroxide was reduced at a catalytic C-PB electrode (Equation (3)). In short, the detection of $H_{2}O_{2}$ is used to quantify the glucose concentration in the solution as the number of electron flow is proportional to the glucose molecules present [28].

(1) $$\mathrm{Glucose}+\mathrm{GOx}-{\mathrm{FAD}}^{+}\to \mathrm{Gluconolactone}+\mathrm{GOx}-{\mathrm{FADH}}_{2}$$

(2) $$\mathrm{GOx}-{\mathrm{FADH}}_{2}+O_{2}\to \mathrm{GOx}-\mathrm{FAD}+H_{2}O_{2}$$

(3) $$\mathrm{KF}e^{\mathrm{III}}[Fe^{\mathrm{II}}{\left(\mathrm{CN}\right)}_{6}]+K^{+}+H_{2}O_{2}+2e^{-}⇌K_{2}Fe^{\mathrm{II}}\left[Fe^{\mathrm{II}}{\left(\mathrm{CN}\right)}_{6}\right]+2OH^{-}$$

Cyclic voltammetry is a common technique used to investigate the reduction and oxidation processes of molecular species. Cyclic voltammetry evaluation is one of the necessary electroanalysis tools to find the optimum peak potential to be used as a parameter in chronoamperometry [29,30]. For the preliminary test, we selected 50 mM glucose solution for evaluation and compared the response with blank (PBS solution) to compare the existence of $H_{2}O_{2}$ (came from the enzymatic reaction of glucose) and no $H_{2}O_{2}$ content (only PBS). We selected a bit high concentration for the consideration that the platform may have a drop of the measurement responses because of the fabric structure that the solution cannot keep sustained on the surface of the electrode on the top layer of the platform, precipitated underneath the fabric layer. Cyclic voltammetry measurements were conducted in the presence of 50 mM glucose and the absence of glucose (using 0.1 M PBS) at scan rates 0.005 V/s to evaluate the optimum detection potential of $H_{2}O_{2}$ detection.

**Figure 5:**
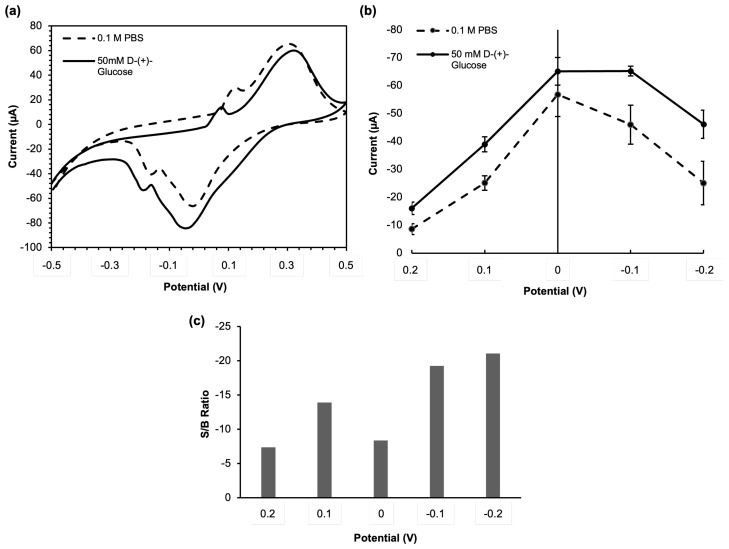
(**a**) CVs of the glucose mFED as obtained in the absence and presence of $H_{2}O_{2}$. (**b**) Effect of applied potential on the glucose mFED extracted from data in part (**a**). (**c**) Signal-to-background ratio extracted from the data shown in part (**b**).

Figure 5a shows the graph comparing the CV responses of PBS and glucose solution. We can see the CV response of PBS in Figure 5a is different from those in Figure 4. We can see clear peak responses when the C-PB-GOx electrode CV tests the PBS solution, showing both anodic and cathodic reactions. However, the response was not shown when the C-PB electrode CV tested PBS solution. From this phenomenon, we can see that the C-PB only show redox reactions when the enzyme is present on it, revealing the assisted/triggered redox reactions of C-PB toward PBS. On the other hand, during glucose measurement, we can see that the redox peaks are shifted. The shifted peaks can be used as evidence that the presence of glucose was successfully detected enzymatically, according to Equations (1)–(3). In addition, it also shows that the GOx is well contained on the electrode and successfully reacted with glucose. The existence of both oxidation and reduction peaks shown on both anodic and cathodic directions thus reflect the reduction reaction of $H_{2}O_{2}$ at PB is reversible with PW oxidation reaction, as shown in Equation (3) [31]. Considering the occurrence of these anodic and cathodic peaks, we need to evaluate the ratio between the peak current and its background current obtained over the important potential range of CV. We select five representative potentials, −0.2, −0.1, 0, 0.1, and 0.2 V to measure this ratio. This strategy is necessary to select which applied potential can give the best response when we later conducted chronoamperometry analysis to calculate the limit of detection.

The ratio between $H_{2}O_{2}$ and background current (S/B ratio) at each potential was analyzed as displayed in Figure 5c. The optimum potential of −0.2 V was chosen for further experimentation because it has the highest ratio between the signal for glucose detection and background current, as depicted in Figure 5c. Peak potential of −0.2 V categorized as a reduction potential and it is in the cathodic direction, thus confirming the capability of the C-PB electrode as the catalytic redox mediator assisting the reduction reaction of $H_{2}O_{2}$ as shown in (Equation (3)) [32]. The higher potential was not investigated to avoid reaction interference from endogenous compounds [33]. The potential of −0.2 V was used for the applied potential for chronoamperometry measurement in glucose sensing application.

#### 3.2.2. Analytical Performance

**Figure 6:**
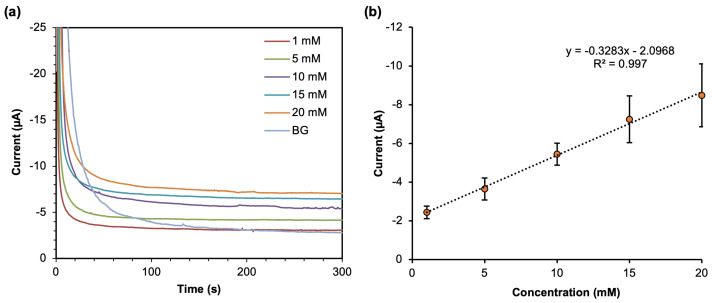
(**a**) Chronoamperograms of glucose with different concentrations ranging from 0–20 mM. (**b**) Linear calibration curve of glucose detection.

After determining the optimum detection potential for $H_{2}O_{2}$, chronoamperometry measurements in different concentrations ranging from 0 to 20 mM were performed with the applied potential of −0.2 V for 300 s (Figure 6a). The anodic current for the last 10 s (the apparent steady-state current) was averaged and used to generate a linear calibration curve (Figure 6b). From the linear calibration curve, it can be deduced that this device has good linearity in the range of 5–20 mM. The linear range of this device covers the clinically relevant range for blood glucose concentration in humans, which is 3.9 to 11 mM. Hypoglycemia condition is categorized if the glucose level is under 3.9 mM (70 $\mathrm{mg}\cdot {\mathrm{dL}}^{-1}$), whereas the normal blood glucose level in humans is between 4.0 and 5.4 mmol/L (72 to 99 $\mathrm{mg}\cdot {\mathrm{dL}}^{-1}$) when fasting and up to 7.8 mmol/L (140 $\mathrm{mg}\cdot {\mathrm{dL}}^{-1}$) at 2 h after eating. Hyperglycemia is when the glucose level is above 7.0 mM (126 $\mathrm{mg}\cdot {\mathrm{dL}}^{-1}$) when fasting and above 11.0 mM (220 $\mathrm{mg}\cdot {\mathrm{dL}}^{-1}$) 2 h after meal [34].

The device’s sensitivity was calculated from the slope of the linear calibration plot, which is 0.3283 μA/mM with the coefficient of determination ($R^{2}$) of 0.997. In addition, from our platform, based on the area of the working electrode inside the circle of the hydrophobic wax (as the sensing area) having approximately 4.6 ${\mathrm{mm}}^{2}$, the calculated sensitivity related to geometric area is 0.0713 $\mu A\cdot \;{\mathrm{mM}}^{-1}\cdot {\mathrm{mm}}^{-2}$.

The limit of detection (LOD) and limit of quantification (LOQ) of the mFED were calculated from the standard deviation of the blank measurement. LOD is the concentration that produced the signal three times the standard deviation of the blank, and LOQ is the concentration that produced the signal ten times the standard deviation of the blank [35]. The LOD and LOQ of this device were found to be 0.98 mM and 3.26 mM, respectively. From the concentration range that we evaluated, we did not observe the typical Michaelis–Menten response on our range of glucose measurement, as the response did not show the saturation response [29,36].

In this preliminary work in developing a fabric-based electrochemical sensor, the large error that occurred during the measurement was considered to be coming from the condition of the analyte solution that was not fully kept on the surface of the electrode, as some portion of the liquid passed through the top layer fabric where the electrode patterns are located. As a response, some fluctuations in the amount of liquids affect the response of the sensor.

#### 3.2.3. Analytical Validation

The device’s clinical performance was also tested using human serum Chemistry Control Level I and Level II. A good correlation was found between the proposed method using the mFED and glucometer in the Chemistry Control Level I (with lower concentration). Although the linear calibration plot of the mFED shows an acceptable dynamic range between 0 and 20 mM of glucose concentration, when tested using diluted Chemistry Control Level II, the device cannot quantify the amount of glucose concentration in the sample accurately (see Table 3).

### 3.3. Continuous Microfluidics Fabric-Based Electrochemical Device

Continuous measurements were performed to demonstrate the capability of continuous mFED to monitor glucose levels in real-time. This device does not need an external pumping apparatus for sample delivery. Because of the cotton fabric’s wicking capability, the sample solution from the reservoir can wick via the capillary of the matrix to reach the reaction zone and then flow out through the outlet. The mFED inlet was dipped into a sample solution, and the outlet was placed on top of cotton fabric to reduce the wicking time. As the sample solution wicked from the reservoir into the sample chamber of the mFED, the sample reacted with the electrodes and produced a corresponding anodic current. Due to the mFED design, which was fabricated with a hydrophobic barrier, the sample solution can flow through the mFED without leaking.

**Figure 7:**
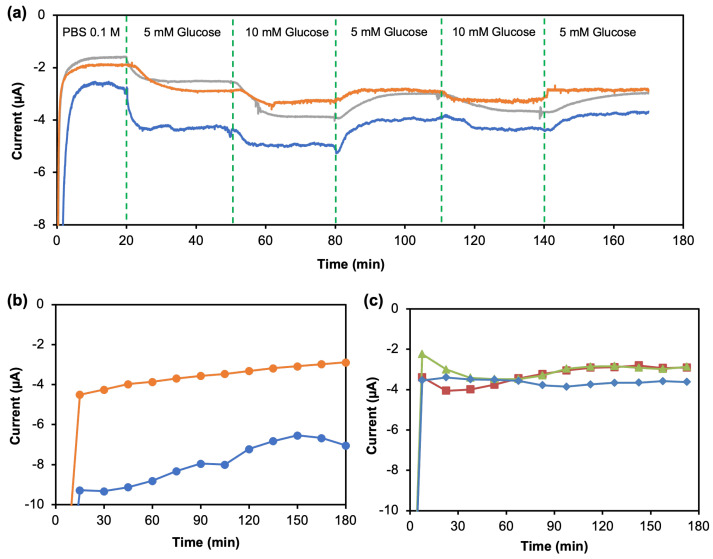
Results on continuous glucose evaluations: (**a**) Chronoamperograms of continuous dynamic measurement of mFED under three different solutions: 0.1 M PBS, five mM glucose, and ten mM glucose. (**b**) Continuous measurement using a real sample (Chemistry Control Level I) for three hours. (**c**) Chronoamperograms of enzyme retention experiment using 2D µFED for three hours using intermittent sampling with 15 min intervals.

As shown in Figure 7a, the amperometric response of the glucose mFED shows a quick response when a new sample is introduced into the inlet. Likewise, the anodic current shows an immediate response. The experiments were repeated using three different devices. The three curves in Figure 7a show a similar pattern to the dynamic measurement. However, each of the curves did not start at the same value. It happened due to the delay for the mFED sensing area to receive a flow of liquid from the subsequent solution, from the inlet in the container to the sensing area created by the circular hydrophobic wax. In our platform, we found that it took approximately 5 min for the chronoamperometry analysis to obtain a stable measurement. Therefore, the system requires precalibration to test their response and determine the offset value. In addition, further calibration is needed because this mFED was produced with a method with a low automation level; hence, a slight difference between each device still exists.

Chronoamperometry was measured for three hours using Chemistry Control Level I as the sample, and the experiment was conducted on two different mFEDs. Data points in Figure 7b were taken every 15 min. However, as the time increases, the anodic current also decreases, not producing a stable result throughout the three hours. The rate of enzymatic reaction decreases as the enzyme concentration on the mFED decreases. The anodic current produced is proportional to the hydrogen peroxide concentration in the enzymatic reaction.

Two hypotheses could be explained regarding the current drift. One of the reasons that can induce the decomposition of $H_{2}O_{2}$ is enzymatic impurities. Impurities in the enzyme solution, such as catalase, will decompose 1.0 micromole of $H_{2}O_{2}$ per minute. If catalase is present in the enzyme solution, it can decrease the concentration of $H_{2}O_{2}$ from 10.3 to 9.3 millimolar. Catalase can catalyze the decomposition of hydrogen peroxide ($H_{2}O_{2}$) into water and oxygen.

The second hypothesis in the decrease in current is caused by the immobilized enzyme flushed out from the reaction chamber to the outlet during three hours of measurement with a constant flowing sample. An enzyme retention experiment was performed on 2D mFED using intermittent sampling to prove the second hypothesis. Chronoamperometry measurements were performed for three hours, and 6 μL of chemistry control level 1 was pipetted with an interval of 15 min. As shown in Figure 7c, the three devices exhibit a stable current throughout the measurement; verify that in the intermittent sampling, mFED has a better enzyme retention capability because the immobilized enzyme and sample are only concentrated in the reaction zone. Unlike in the continuous mFED (Figure 7c), due to an additional inlet and outlet for sample delivery, the enzyme immobilized in the reaction zone tends to flush out to the outlet during three hours of measurement using a flowing sample, hence causing the current drift.

### 3.4. Mechanical Deformation Test

To study the effect of mechanical strain on the electrochemistry performance of the continuous mFED, chronoamperometry measurements were done on the mFED under different conditions: normal flat, bending, and folding movement. The behavior of the mFED’s electrochemical performance was studied under mechanical strain to assess its applicability as a wearable sensor. One of the main requirements of a wearable sensor is robustness, the resilience to mechanical strain. The mechanical strain given to the mFED is intended to mimic the strain when the mFED is integrated into clothing. For this experiment, chronoamperometry was measured for 360 min (6 h) using a five mM glucose solution as the sample. The experiment was conducted using a beam method [37] attached to a two-axis linear stage. One side of the mFED was clamped in position, and the free end of the mFED was attached with calibration mass and put on top of an analytical balance. The calculation of strains from two different loads was described in the Appendix B.

**Figure 8:**
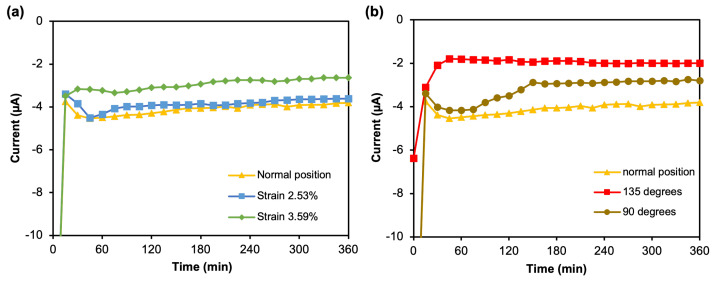
(**a**) The anodic current responses of the 3D mFED during normal (planar) position and under mechanical strain using flowing 5 mM glucose solution in the reservoir. (**b**) The anodic current response of the 3D mFED during normal (planar) position and under different folding conditions using a flowing five mM glucose solution in the reservoir.

Figure 8a shows the chronoamperograms of the mFED under several bending strains. There was no major stability difference in the mFED between different strain values observed from the graph. Although there was a slight current drift (decline) over time, the anodic response still exhibits a stable current from minutes 50 to 360, considering it was under mechanical strain. Under strain 3.59%, with twice the load value than 2.53%, the current response of the mFED differs 2 μA over the normal position. However, the experiment proved the stability and the duration of usability of the 3D mFED to do continuous monitoring, reaching 6 h. The mFED can perform a continuous electrochemical measurement for a long time due to the device’s compact design. In addition, effective wax transfer onto the cotton fabrics increases the robustness of the device itself. The hydrophobic barrier made by wax patterning prevents any leakage of the immobilized enzyme and the sample solution from the reaction zone into the surrounding substrate. The next experiment was evaluating mFED through platform deformation at 135° and 90° folding positions (Figure 8b). The experimental protocol for chronoamperometry measurement used a similar method as the previous experiment. The result shows that the anodic current response for the 3D mFED during normal position and 135° shows a stable signal from minutes 50 to 350. When subjected to a 90° angle, the chronoamperometric response decreased at the beginning of measurement and reached a stable response in min 150 to 350. From these experiments, we see a tendency that our system is still relatively stable during bending conditions. Nonetheless, further mechanical evaluations are needed in the future to evaluate the trends of the response under a certain range of mechanical strains and the responses under movement conditions.

For future work, we consider that the measurement can be improved when the base of the fabric gives enough hydrophobicity to hold the analyte solution to be measured on the surface where the electrodes are located and do not necessarily need to create multi-folded layers for creating well. One of the good ideas is creating or selecting a double-sided hydrophobic/hydrophilic fabric, where the hydrophilic surface is located on the same side of electrodes, and the hydrophobic surface is located on the opposite side [38]. This strategy promotes straightforward solutions and one-step manufacturing.

## 4. Conclusions

The electrode paste transfer method was optimized using the stencil printing method. Using the stencil printing method, the production of μFED can be more efficient, producing a functioning mFED with a high success rate. In addition, the quantitative measurement of blood glucose was performed in this research. The mFED has a linear working range of 0–20 mM of glucose, with the device’s sensitivity of 0.3283 $\mu A\cdot \;{\mathrm{mM}}^{-1}\cdot {\mathrm{mm}}^{-2}$ and LOD and LOQ of 0.98 mM and 3.26 mM, respectively. Due to the wicking capability of cotton fabrics, the 3D mFED can perform continuous dynamic measurements for real-time monitoring without any form of external force. The feasibility was demonstrated using a real sample of human serum. Mechanical deformation only has a slight impact on the performance of the continuous measurement of the 3D device.

## Figures and Tables

**Table 1:** Resistance comparison between brushing and stencil printing method (* SD *n* = 20).

Electrode	Resistance ± SD * (Ω)	
	Brush	Stencil Printing
Reference electrode	44.66±103.55	1.13±0.30
Working electrode	15,748.45±23,936.78	255.03±46.99
Counter electrode	20,117.25±21,350.73	434.80±59.17

**Table 2:** Comparison of electrode paste transfer method.

Parameter	Method	
	Brush	Stencil Printing
Difficulty	Very easy	Easy
Time	Time-consuming	Time-saving
Electrode paste amount	Low	High
Resistance value	High	Low
Standard deviation	High	Low

**Table 3:** Device testing results using real sample.

Electrode	Concentration (mM ± sd)			
	Chemistry Control Level I		Chemistry Control Level II	
	Glucometer	mFED	Glucometer	mFED
Glucose	7.30±0.21	7.85±1.37	14.13±0.56	8.27±0.32
Percent Error (%)	7.00		41.47	

## Data Availability

Not applicable.

## Abbreviations

The following abbreviations are used in this manuscript:

mFED	Millifluidic fabric-based electrochemical device
LOD	Limit of detection
LOQ	Limit of quantification
FED	Fabric-based electrochemical device
PB	Prussian blue
PW	Prussian white
PBS	Phosphate-buffered saline
WE	Working electrode
RE	Reference electrode
CE	Counter electrode
C-PB	Carbon-Prussian blue
S/B ratio	Signal-to-background ratio

## Appendix A. Calculation of Mechanical Deformation Test

The strain was calculated from the deflection and material properties of cotton fabric using the following equation:

(A1) $$\sigma_{s}=\frac{My}{I}$$

where $\sigma_{s}$, *M*, *y*, and *I* are stress at the surface, moment, distance from neutral axis (half of the thickness), and moment of inertia, respectively. The strain also can be expressed from Equation (2), while the moment of inertia is shown in Equation (3) below:

(A2) $$I=\frac{WH^{3}}{12}$$

(A3) $$\sigma_{s}=\frac{6FL}{WH^{2}}.$$

*W*, *H*, *F*, and *L* are width, thickness, load, and length, respectively. To estimate Young’s modulus of the mFED, we follow the work from Liu et al. [37], and the equations below were used:

(A4) $$E=\frac{4FL^{3}}{\delta WH^{3}}$$

(A5) $$\epsilon =\frac{\sigma }{E}.$$

*E*, δ, and ϵ are Young’s modulus, deflection, and strain. Finally, the strain was calculated from the following equation:

(A6) $$\epsilon =\frac{3\delta H}{2L^{2}}$$

## Appendix B. Results on Mechanical Deformation Test

The strain given to mFED can be calculated from the deflection point and characteristic of the mFED itself.

### Appendix B.1. Deflection Point 1

(A7)
$$\begin{array}{cc} \sigma_{s} & =\frac{6FL}{WH^{2}}\\ & =\frac{6(248.88\;\mathrm{Nm})(9.5\;\mathrm{mm})}{\left(19\;\mathrm{mm}\right){\left(1\;\mathrm{mm}\right)}^{2}}\\ \; & =746.64\;{\mathrm{mN}/\mathrm{mm}}^{2} \end{array}$$

(A8)
$$\begin{array}{cc} E & =\frac{4FL^{3}}{\delta WH^{3}}\\ \; & =\frac{4\left(248.88\;\mathrm{Nm}\right){\left(17.5\;\mathrm{mm}\right)}^{3}}{\left(9.5\;\mathrm{mm}\right)\left(19\;\mathrm{mm}\right){\left(1\;\mathrm{mm}\right)}^{3}}\\ \; & =29558.77\;\mathrm{kPa} \end{array}$$

(A9)
$$\begin{array}{cc} \epsilon & =\frac{\sigma }{E}\\ \; & =\frac{746.64\;{\mathrm{mN}/\mathrm{mm}}^{2}}{29558.77\;\mathrm{kPa}}\\ \; & =0.0253=2.53\% \end{array}$$

### Appendix B.2. Deflection Point 2

(A10)
$$\begin{array}{cc} \sigma_{s} & =\frac{6FL}{WH^{2}}\\ \; & =\frac{6(494.13\;\mathrm{Nm})(9.5\;\mathrm{mm})}{\left(19\;\mathrm{mm}\right){\left(1\;\mathrm{mm}\right)}^{2}}\\ \; & =1482.39\;{\mathrm{mN}/\mathrm{mm}}^{2} \end{array}$$

(A11)
$$\begin{array}{cc} E & =\frac{4FL^{3}}{\delta WH^{3}}\\ \; & =\frac{4\left(494.13\;\mathrm{Nm}\right){\left(17.5\;\mathrm{mm}\right)}^{3}}{\left(13.5\;\mathrm{mm}\right)\left(19\;\mathrm{mm}\right){\left(1\;\mathrm{mm}\right)}^{3}}\\ \; & =41297.88\;\mathrm{kPa} \end{array}$$

(A12)
$$\begin{array}{cc} \epsilon & =\frac{\sigma }{E}\\ \; & =\frac{1482.39\;{\mathrm{mN}/\mathrm{mm}}^{2}}{41297.88\;\mathrm{kPa}}\\ \; & =0.0359=3.59\% \end{array}$$

## Appendix C. Measurement Setup of Continuous Chronoamperometry

Photograph of continuous chronoamperometry measurement setup showing the inlet of mFED was dipped into a reservoir containing sample solution. There are three solutions that were prepared: PBS 0.1 M, glucose 5 mM, and glucose 10 mM.
